# In Vitro and In Vivo Efficacy of DNA Damage Repair Inhibitor Veliparib in Combination with Artesunate against *Echinococcus granulosus*

**DOI:** 10.1155/2020/8259820

**Published:** 2020-07-01

**Authors:** Y. F. Li, L. M. Wen, J. Zhao, G. D. Lv, S. Lu, S. Lu, X. Zheng, B. Chen, C. Y. Tian, Y. H. Gong, H. J. Gao, J. H. Wang

**Affiliations:** ^1^Pharmaceutical Department, The First Affiliated Hospital of Xinjiang Medical University, Urumqi 830054, China; ^2^College of Pharmaceutical Sciences, Xinjiang Medical University, Urumqi 830054, China; ^3^State Key Laboratory of Pathogenesis, Prevention, Treatment of Central Asian High Incidence Diseases, Urumqi 830054, China; ^4^Clinical Pharmacy, The Third Affiliated Hospital of Bengbu Medical College, Suzhou 234000, China; ^5^Department of Orthopedics, The First Affiliated Hospital of Xinjiang Medical University, Urumqi 830054, China

## Abstract

Cystic echinococcosis (CE), caused by the cestode *Echinococcus granulosus*, is a worldwide chronic zoonosis. Albendazole (ABZ) and mebendazole are effective against CE, but a high dosage in a long-term period is usually required. In this study, we evaluate the effects of DNA damage repair inhibitor (i.e., Veliparib) in combination with artesunate (AS) on hydatid cysts. For the *in vitro* assay, protoscoleces of *E. granulosus* (*E.g* PSCs) were incubated with low AS (AS-L, 65 *μ*M), moderate AS (AS-M, 130 *μ*M), and high AS (AS-H, 325 *μ*M), AS-L/M/H+Veliparib (10 *μ*M), and ABZ (25 *μ*M), respectively. The AS-H+Veliparib group showed the maximal protoscolicidal effects. Ultrastructural change revealed that germinal layer (GL) cells were reduced, and lipid droplets appeared. AS could induce DNA injuries in PSCs. The 8-OHdG was expressed in the PSCs and GL of the cysts in mice, especially in the presence of Veliparib. The most severe DNA damages were observed in the AS-H+Veliparib group. Meanwhile, the expression of ribosomal protein S9 (RPS9) gene in the AS-H+Veliparib group was significantly lower than that in the AS-H group. The *in vivo* chemotherapeutic effects of AS-L (50 mg/kg), AS-H (200 mg/kg), and AS-H+Veliparib (25 mg/kg) were assessed in experimentally infected mice. Upon 6 weeks of oral administration, ultrasonography was used to monitor the volume change of vesicles. Maximum potentiation was seen on day 15 with values (versus AS) of 34 (*P* < 0.05) for AS-H + Veliparib. It led to the reduction of cyst weight (55.40%) compared with the model group (*P* < 0.01), which was better than AS alone (52.84%) and ABZ-treated mice (55.35%). Analysis of cysts collected from AS-H+Veliparib-treated mice by transmission electron microscopy revealed a drug-induced structural destruction. The structural integrity of the germinal layer was lost, and the majority of the microtriches disappeared. In conclusion, our study demonstrates that AS or AS in combination with Veliparib is effective for treating CE, especially the combination group. On this basis, AS represented promising drug candidates in anti-CE chemotherapy.

## 1. Introduction

Cystic echinococcosis (CE), also known as cystic hydatid disease, is a zoonotic infection caused by larval stage of *Echinococcus granulosus* [1, 2]. Nowadays, benzimidazole compounds, particularly the albendazole (ABZ), have been reported to be effective against CE [3]. However, these compounds, with poor water solubility, were classified as type II drugs on the Biopharmaceutical Classification System [4]. The low dissolution rate of ABZ triggers insufficient absorption in the gastrointestinal tract, resulting in a low plasma drug level [5]. Moreover, long-term medication of ABZ results in severe adverse events in these patients. Therefore, novel chemotherapeutic agents with good efficacy are urgently required.

Artemisinin, an ancient Chinese herbal, was initially isolated in 1972 from *Artemisia annua*. L. The World Health Organization (WHO) recommends the artemisinin-based combination therapy (ACT) as the first-line treatment for *Plasmodium falciparum* infection [6]. In recent years, artemisinin and its derivatives have been reported to show curative effects on protozoans [7], nematodes [8], trematodes, and tumor [9]. These findings prompted us to investigate the potency of artemisinin for treating CE. Artemisinin and its derivatives including artesunate (AS) showed high efficiency with low toxicities. The effects of AS were further evaluated using the BALB/c mouse model [10, 11]. AS combined with ABZ resulted in obvious reduction of the cyst weights compared to single application of AS [12]. However, the main mechanisms of AS against parasites are still not clear.

The modulatory effects of AS on the parasite transcriptome were mainly associated with the regulation of protein involved in encoding companion, transcriptional activation, proteasome, oxidative stress, and cell cycles [13]. Parasite exposure to AS resulted in a rapid depolarization of parasite *ΔΨ*m and *ΔΨ*p, which was shown to be mediated via the generation of ROS initiated by iron bioactivation of endoperoxides and/or catalysed by iron-dependent oxidative stress [14]. Therefore, we speculated that the main pharmacological roles of artemisinin were associated with the generation of free radicals by the endoperoxide bridge and induction of oxidative stress, which ultimately led to parasitic death [15, 16]. In our previous study, 15 genes associated with the genetic processing were upregulated after artemisinin treatment, including DNA replication, repair, transcription, and translation [17]. Meanwhile, Wen et al. [18] firstly cloned the *RPS9* gene that participated in DNA repair and developmental regulations from the *E. granulosus* protoscoleces under oxidative stress induced by AS.

DNA repairing mechanism is usually activated upon DNA damages. Efficient DNA repair is an important mechanism for resistance. Therefore, inhibition of DNA repair would be the most effective for this process [19, 20]. The oxidative stress-induced DNA damages were mainly repaired by base excision repair (BER) [21] that involved the base pair deletion and generation of 8-OHdG [22]. As an important component of the BER pathway, poly (ADP-ribose) polymerase (PARP) is a multifunctional protein involved in the activation of the BER downstream repairing protein recruiting the DNA polymerase and ligase. Besides, it closely mediated the DNA damage recognition and signal transmission. Among the PARP family, PARP-1 is the extensively studied [23]. In the early-stage clinical screening, Veliparib (ABT-888) was considered the third-generation inhibitor of PARP-1, with high activity and selectivity, exerting higher efficiency in combination with other agents [24].

In this study, we aim to investigate the efficiency of Veliparib combined with AS on treating CE. For the *in vitro* experiments, eosin staining and alkaline phosphatase activity assay were utilized to detect protoscoleces (PSCs) activity. The ultrastructural change of PSCs were observed by transmission electron microscopy (TEM). For the *in vivo* assay, ultrasonography was used to monitor the volume change of vesicles, and calculate the cyst inhibition rate. TEM was used to evaluate the efficiency of AS and the combination of AS and Veliparib on treating CE.

## 2. Materials and Methods

### 2.1. *In Vitro* Culture of PSCs

PSCs were obtained from hydatid cysts of naturally infected sheep livers that were >collected from abattoirs in Urumqi, Xinjiang Uyghur Autonomous Region. PSCs were digested using 1% of pepsin in distilled water at pH 2.0 for 30 min. Cyst culture was performed under aseptic conditions according to our previous description [18].

### 2.2. *In Vitro* Drug Treatment of PSCs

PSCs were seeded in 96-well cell culture plates in 0.2 mL cultivation medium at 37°C with 5% CO_2_. The PSCs were divided into the following groups: (a) DMSO group, treatment with 1% (*v*/*v*) DMSO; (b) Veliparib group, treatment with 10 *μ*M Veliparib; (c) ABZ group, treatment with 50 *μ*M ABZ, serving as a positive control; (iv) AS-L group, treatment with 65 *μ*M AS; (d) AS-M group, treatment with 130 *μ*M AS; (e) AS-H group, treatment with 325 *μ*M AS; (f) ABZ+Veliparib group, treatment with 50 *μ*M ABZ and 10 *μ*M Veliparib; (g) AS-L + Veliparib group, treatment with 65 *μ*M AS and 10 *μ*M Veliparib; (h) AS-M+Veliparib group, treatment with 130 *μ*M AS and 10 *μ*M Veliparib; (i) and AS-H+Veliparib group, treatment with 325 *μ*M AS and 10 *μ*M Veliparib. In the AS combined with Veliparib groups, Veliparib was added about 30 min before AS. AS (B20992) and ABZ (A822534-50) were purchased from Yuanye Biotechnology (Shanghai, China). Veliparib (S1004c), purchased from Selleck Chemical (Shanghai, China), was dissolved in DMSO for subsequent analysis. After 4 days of drug treatment, PSCs were collected every day, and each experiment was performed at least in triplicate.

### 2.3. Eosin Staining and ALP Determination

PSCs viability was assessed by 1% Eosin staining followed by morphologic observation. The corresponding numbers of viable/nonviable PSCs were determined. We determined the activity of *E. granulosus* alkaline phosphatase (EgALP) in culture medium supernatants of PSCs. During the 4-day treatment, 50 *μ*L culture supernatant was collected daily and stored at -80°C to measure EgALP activity [25] using the commercial alkaline phosphatase (ALP) Assay Kit (Beyotime Biotech, Shanghai, China). After drug treatment, the treated PSCs were collected, and TEM was used to observe the PSCs as described previously [26]. Micrographs were taken on a JEM-100CX II system.

### 2.4. Comet Assay

In the comet assay, PSCs were divided into the following groups: DMSO group, treatment with 1% DMSO; ABZ group, treatment with 50 *μ*M ABZ; Veliparib group, treatment with 10 *μ*M Veliparib; AS-L group, treatment with 65 *μ*M AS; AS-H group, treatment with 325 *μ*M AS; and AS-H+Veliparib group, treatment with 325 *μ*M AS and 10 *μ*M Veliparib. After culture for 4 days, the PSCs were collected for comet assay according to the method proposed by Singh et al. [27], with some minor modifications. The microscope slides were immerged with 0.6% agarose, followed by incubating at 65°C overnight. The PSCs culture solution was removed and then was washed using PBS. Then, centrifugation was performed; 90 *μ*L of 0.75% agarose was used to resuspend the PSCs onto the first layer of agarose. After agarose solidification at 4°C for 5 min, the coverslips were removed and the slides were immersed for 4 h at 4°C in lysis solution freshly prepared (2.5 M NaCl, 100 mM Na_2_EDTA, and 10 mM Tris-HCl) containing 1% Triton X-100 and 10% dimethyl sulfoxide. The slides were equilibrated in alkaline solution (1 mM Na_2_EDTA, 300 mM NaOH, pH 13) for 20 min. Electrophoresis was carried out for 20 min at 25 V and 0.83 V/cm. After this, slides were neutralized by washing with 0.4 M Tris-HCl buffer (pH 7.5) for 15 min. Slides were stained with 60 *μ*L propidium iodide solution in the dark.

The images were observed under a magnification of 200x using a fluorescent microscope (Olympus IX73, Japan), and the image for PSCs was acquired using the CASP software. Finally, PSCs were analyzed using the percentage tail DNA of comets [28].

### 2.5. Determination of DNA Damages in *E*.*g* PSCs

Immunodetection of 8-OHdG adducts conducted as previously described [29] with some modifications. The DMSO (1%, *v*/*v*), ABZ (50 *μ*M), Veliparib (10 *μ*M), AS-L/H (65 *μ*M, 325 *μ*M), and AS-H+Veliparib-treated PSCs for 4 days were fixed in 4% paraformaldehyde for 24 h at 4°C. After dehydration, sections were incubated at room temperature for 10 min in 3% H_2_O_2_ and washed three times in PBS. Slides were placed in a container with citrate buffer, followed by heating in a microwave oven at about 98°C. After cooling at room temperature, the slides were blocked with normal goat serum at 37°C for 20 min and incubated with monoclonal mouse antibodies anti 8-OHdG (1 : 100, no. ab62623, Abcam, USA) overnight at 4°C. Subsequently, the mixture was incubated with the secondary goat anti-mouse FITC antibody (1 : 400, no. ab6785, Abcam, USA). Sections were washed three times and with Hoechst 33258 staining. Afterwards, slides were mounted in visualized in a fluorescent microscope (Olympus IX73, Japan), and then, the images of nuclei presenting DNA damage were obtained.

### 2.6. Quantitative Real-Time PCR

PSCs were exposed to cultivation medium, ABZ (50 *μ*M), Veliparib (10 *μ*M), AS-L/H (AS-L, 65 *μ*M; AS-H, 325 *μ*M), and AS-H+Veliparib. After 4 days of drug treatment, total RNA was extracted from freshly isolated PSCs using Trizol reagent (Invitrogen, USA); cDNA was synthesized according to our previous description [17]. For the identification of *EgRPS9* mRNA expression, real-time PCR reaction was carried out as previously described [17].

### 2.7. Western Blot Analysis

Proteins extracted from PSCs were subjected to SDS-PAGE. Separated proteins were electrophoretically transferred to polyvinylidene difluoride (PVDF) membranes (Merck Millipore, Darmstadt, Germany) and immunoblotted with a rabbit anti-RPS9 antibody (ab74711, Abcam, Cambridge, UK). A rabbit anti-*β*-actin antibody (ab205718, Abcam) served as the loading control. Immunoreactive proteins were visualized using the Odyssey Infrared Imaging System (Li-Cor, Lincoln, Nebraska) as described by the manufacturer. The relative intensities of the bands were quantified by densitometric analysis. The densitometric plots of the results were normalized to the intensity of the *β*-actin band.

### 2.8. *In Vivo* Treatment with Compounds

The animal handling was conducted with the approval of the Institutional Animal Care and Use Committee, the First Affiliated Hospital of Xinjiang Medical University (No. IACUC-20150225-70). Pathogen-free female Kunming mice (6-8 weeks) were purchased from Xinjiang Medical University Animal Experiment Center and raised in the animal facility of the First Affiliated Hospital of Xinjiang Medical University.

For the infection challenge of mice, PSCs were precultured *in vitro* to generate small cysts (microcysts, 200-300 *μ*m in diameter) [30]. Each mouse was intraperitoneally transplanted with 25 microcysts suspended in 0.3 mL RPMI 1640 medium. About 180 days postinfection, the mice were randomly divided into seven groups including (a) the control group, normal mice with no infection challenge (*n* = 10); (b) model group, PSC-infected mice that received 500 *μ*L of 0.4% CMC-Na via lavage; (c) Veliparib group, PSC-infected mice that received Veliparib (25 mg/kg) dissolved in 0.9% NaCl solution (pH 4.0); (d) ABZ group, PSC-infected mice that received ABZ with a concentration of 200 mg/kg via lavage; (e) AS-L group, PSC-infected mice that received 50 mg/kg AS via lavage; (f) AS-H group, PSC-infected mice received 200 mg/kg AS via lavage; and (g) AS-H+Veliparib group, PSC-infected mice that received Veliparib (25 mg/kg) combined with 200 mg/kg AS. The lavage of AS-H group lasted for 6 weeks. The size of the cysts in mice was determined using ultrasonography according to the formula of (length × width^2^)/2. The volume growth rate of cysts was calculated as follows: T/C% = (mean cyst volume in the test group/mean cyst volume in the control group) × 100%.

The cysts were separated after treatment, followed by weighing the cyst. Then the wet weight of cysts and cyst-growth inhibiting rate were calculated using the following formula: cyst growth inhibiting rate = (wet weight of cyst in the control − wet weight of cyst in the treatment group)/wet weight of cyst in the model control × 100%.

### 2.9. TEM

For probing tissue changes after drug treatments, cysts were processed for TEM analysis as described previously [26]. Micrographs were taken on a JEM-100CX II TEM.

### 2.10. Statistical Analysis

All data were shown as the mean ± standard deviation. SPSS 19.0 software was utilized for the data analysis. The univariate analysis of variance was used to compare the viability rate of PSCs between the experimental groups *in vitro*. Student's *t*-test and chi square test were used to analyze the comparison. *P* < 0.05 was considered to be statistically significant.

## 3. Results

### 3.1. Effects of Drugs on the PSCs

**Figure 1:**
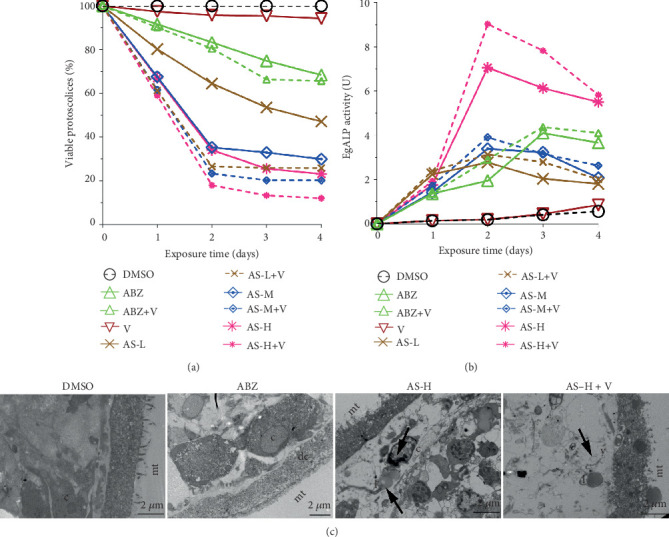
Viable rate of *E. granulosus* PSCs, activity of alkaline phosphatase in culture supernatant, and TEM analyses of drug-treated *in vitro*. PSCs were treated with DMSO, Veliparib (10 *μ*M), ABZ (25 *μ*M), AS-L (65 *μ*M), AS-M (130 *μ*M), AS-H (325 *μ*M), and AS-L/M/H+Veliparib (10 *μ*M). (a) Viability was determined through 1% eosin staining. (b) EgALP activity was measured in culture supernatants at different time points. (c) TEM analyses of *E.g* PSCs and DMSO group showed clear and visible cells, more and neat microtriches protruding from the tegument walls, and a normal germinal layer. mt: microtriches; c: parenchyma cell. Representative micrographs of protoscoleces after treatment with ABZ, AS-L/M/H, and AS-L/M/H+Veliparib on day 4. l: lipid droplets; v: vacuoles; dc: appearance of small vacuoles in the distal cytoplasm; DMSO: dimethyl sulfoxide; V: Veliparib.

PSCs were collected on days 1, 2, 3, and 4 after treatment. On day 4, the viability of PSCs in the DMSO group and Veliparib group was higher with a ratio of 100% and 94.27 ± 1.19%, respectively (Figure 1(a)). The death rate of PSCs in the AS plus Veliparib group showed obvious increase compared with that of the single AS group (*χ*^2^ = 104.155, 24.748, and 34.016; *P* < 0.01). This indicated that AS combined with Veliparib was superior to the AS group in killing the PSCs.

The activity of ALP in AS groups and the combination of AS and Veliparib group in PSCs showed a significant increase after treatment, which was in a time-dependent manner especially in the AS-H group and AS-H+Veliparib group (Figure 1(b)). On day 2, it reached the peak level, followed by a gradual decline. In the AS-H group or AS-H combined groups, the increase of ALP was the most obvious, which reached the peak level on day 3 in ABZ group. The activity of ALP in the combination of AS and Veliparib was more superior than that of the AS group.

The ultrastructural images of TEM are shown in Figure 1(c). DMSO and Veliparib-treated PSCs showed no ultrastructural alteration in PSCs tissue. There were many microvillus in the outer layer. The cell has a complete morphological structure, and the nuclear structure was dense. The villus in the outer layer in the ABZ group was no longer available, and the internal structure was not regular. The cellular structures were completely destroyed. AS-H treatment showed disorder or even loss of microtriches, together with the presence of abnormalities in internal structure, lipid droplets, scattered nucleoli, and heterochromatin margination phenomenon. AS-H plus Veliparib showed an even more dramatic effect, which resulted in complete destruction of the PSCs tissues. The structural integrity of the germinal layer was totally lost, and the majority of the microtriches were absent. Besides, the distal cytoplasm was loose, together with the reduction of germinal layers. The lipid droplets and vacuoles were available. The results of ultrastructure observation were consistent with those of PSCs mortality and ALP activity.

### 3.2. Comet Assay in *E.g* PSCs

**Figure 2:**
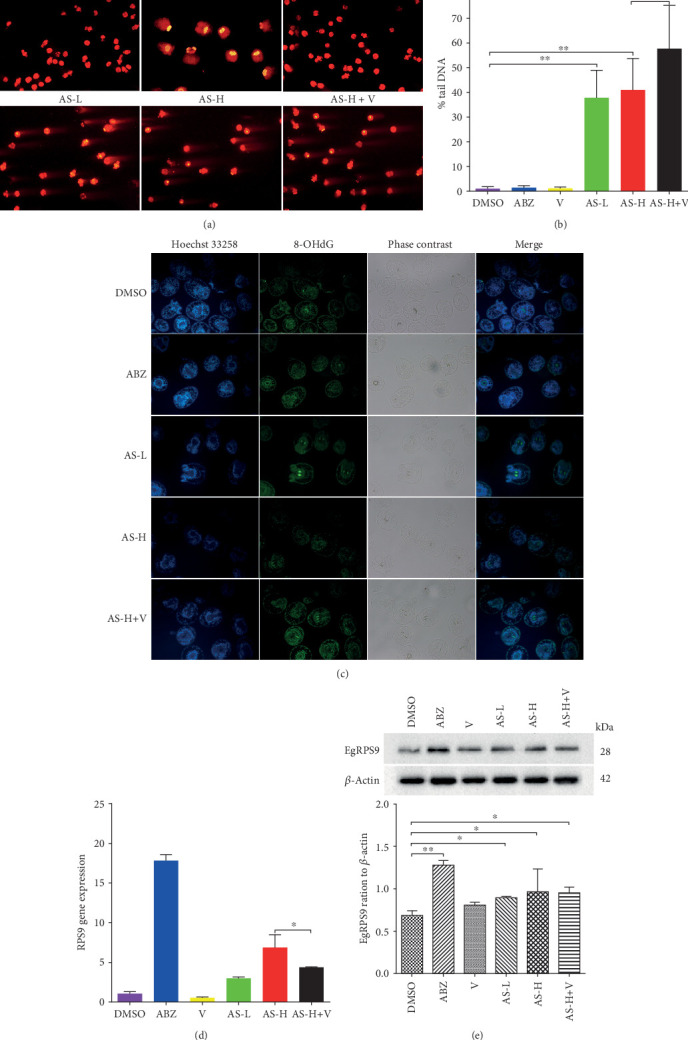
Correlation index detection in each group after the drug intervention. (a) The comet images of *E.g* PSCs on DNA damage. (b) Percentage tail DNA of comet value assay; ^∗^*P* < 0.05; ^∗∗^*P* < 0.01. (c) Oxidative damage products of DNA in PSCs exposed to different concentrations of drugs *in vitro*, as determined by 8-OHdG immunolocalization. PSCs were treated with DMSO, ABZ (25 *μ*M), AS-L (65 *μ*M), AS-H (325 *μ*M), and AS-H (325 *μ*M)+Veliparib (10 *μ*M) for 4 days. Hoechst was used for nuclear staining. Positive 8-OHdG nuclei, green color. (d) The gene expression levels of *EgRPS9* in PSCs were evaluated by qRT-PCR and Western blot (e). DMSO: dimethyl sulfoxide; V: Veliparib.

The DNA damages as revealed by comet assay are shown in Figure 2(a). The percentage of DNA in the tail value is shown in Figure 2(b). The morphology in the DMSO group, Veliparib group, and ABZ group was normal, with no tail formation. Compared with the Veliparib and DMSO groups, significant differences were noticed in the percentage of tail DNA values in the AS groups (*F* = 62.270, *P* < 0.01). Compared with the AS-H group, differences were observed in the percentage of tail DNA values in the AS-H + Veliparib group (*t* = 2.491, *P* < 0.05). Therefore, the tailing in the AS-H+Veliparib group showed higher DNA percentage in tail values, indicating the DNA damages were more severe in these groups.

### 3.3. Oxidative DNA Damage in *E*.*g* PSCs

The expression of 8-OHdG in the PSCs of each group is shown in Figure 2(c). There was a gradual increase in the number of positive nuclei for 8-OHdG (in green); upon treating with Veliparib, the expression of 8-OHdG showed a significant increase compared with that of the AS-H group, indicative of oxidative DNA damage resulting from treatment with increased levels of AS and the combination of AS. Fluorescence was not detected in histological sections of PSCs treated with DMSO, Veliparib, and weak fluorescence with ABZ. PSCs presenting tegument damages (AS-H+Veliparib group) showed higher levels DNA damage in the AS-H+Veliparib group.

### 3.4. Analysis of *EgRPS9* Expression in PSCs

The *EgRPS9* expression level in the ABZ-treated group was 17.28-fold higher than the level in the DMSO group (*P* <0.01, Figure 2(d)). The *EgRPS9* expression levels in the AS-L-treated group and the AS-H treated group were 2.84-fold and 6.66-fold higher compared with that in the DMSO group, respectively (*P* < 0.05, Figure 2(d)). *EgRPS9* expression level in the high AS-dose treated group was about 1.59-fold than that of the AS-H+Veliparib-treated group (*P* < 0.05).

### 3.5. *EgRPS9* Identification by Western Blot

*EgRPS9* protein of the drug-treated groups showed an increase compared with that of the DMSO group (Figure 2(e)), especially that of the ABZ group (*P* < 0.01). In the AS-H+Veliparib group, *EgRPS9* was downregulated compared with that in the AS-H group despite no statistical difference (*P* > 0.05).

### 3.6. AS and Veliparib Regulated the Growth Rate of Cysts in Infected Mice

**Figure 3:**
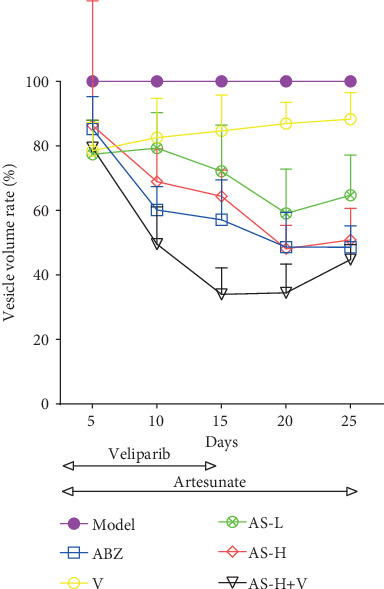
Changes of cyst volume in experimentally infected mice by ultrasonography. The vesicles growth rate was assessed by determining T/C %. The model group received CMC-Na, and treatment groups were given Veliparib (25 mg/kg/d, p.o., q.d.). On days 1-14, AS-H (200 mg/kg/d) was administered 2 h after Veliparib. On days 1-30, ABZ (200 mg/kg/d), AS-L (50 mg/kg/d), and AS-H (200 mg/kg/d, p.o., q.d.) were administered.

Figure 3 shows the growth rate of the cystic volume. Within the 14 days, the growth rate in the AS groups showed a tendency of decline. The decline in the AS combined with the Veliparib group was more obvious than that in the other AS groups. Veliparib could enhance the activity of AS, which was presented by a maximal decline on day 15 in the AS-H+Veliparib group with the minimal growth rate.

**Figure 4:**
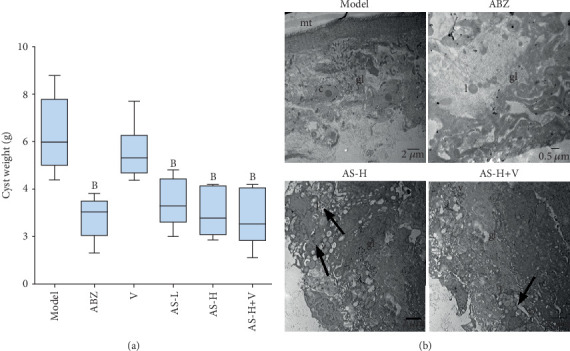
*In vivo* efficacy response of Veliparib in combination with AS in cystic echinococcosis models. (a) After euthanasia, the cysts were resected and weighed. Data consists of 10 mice per treatment group; bars, SE. For statistical analysis and being compared with the model group, ^B^*P* < 0.01. (b) TEM analyses of the cystic wall after *in vivo* treatment with ABZ, AS-H, and AS-H+Veliparib. The CMC-treated cystic walls showed that the germinal layer (gl) and clearly visible cells were undifferentiated cells (c) and the microtriches (mt) protruded from the tegument wall to laminated layer. Representative micrographs of the cystic wall collected from the infected Kunming mice after the daily lavage of ABZ (200 mg/kg/d), AS-H (200 mg/kg/d), and AS-H (200 mg/kg/d)+Veliparib (25 mg/kg/d). l: lipid droplets; v: vacuoles; V: Veliparib.

ABZ, AS-L/H, and AS-H+Veliparib induced significant reduction in the cyst weights compared with the model group (*P* < 0.01, Figure 4(a)). The mice treated with AS (200 mg/kg/d) in combination with Veliparib exhibited reduction in cyst weight, which was higher than that of the AS group (55.40% vs. 52.84%) and ABZ-treated mice (55.40% vs. 55.35%). However, despite the improved mean efficacy of AS, there was no statistical difference in the cyst weight between the ABZ and AS alone or combination-treated groups.

### 3.7. AS and Veliparib Induce Severe Ultrastructural Change on the Cystic Wall

Figure 4(b) indicates the ultrastructure of the cystic wall by TEM. The corneum layer and germinal layer of the cystic wall in the model group and Veliparib group were clear and normal. There was a large amount of microvillus, and the arrangement was regular. The nucleus was clearly displayed. In the ABZ group, the microvillus was no longer available, and the structure of the germinal layer was not clearly displayed. The amount of cells showed a decline, and the lipid droplet was observed. In AS-H group, the microvillus was no longer available, and the number of cells in the germinal layer showed decline, together with the presence of lipid droplet and vacuoles appeared. In the AS-H+Veliparib group, the microvillus was not available, and the structure of the germinal layer was porous and the texture was not regular. The cells were no longer available. Meanwhile, there were abundant lipid droplet and vacuoles.

### 3.8. Oxidative DNA Damage in Cysts Exposed to Different Concentrations of Drugs *In Vivo*

**Figure 5:**
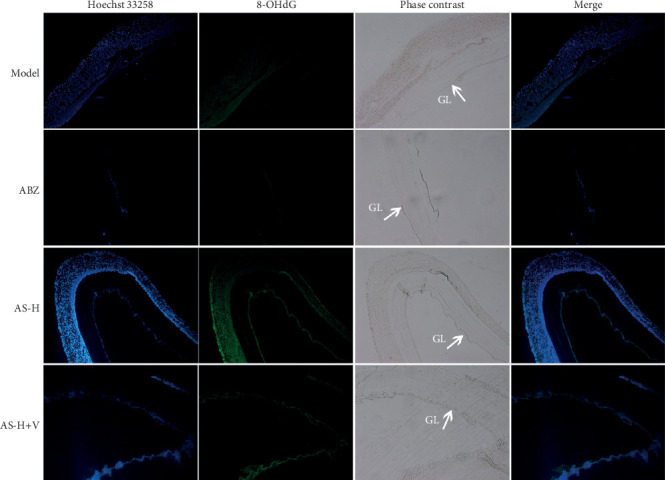
Oxidative DNA damage in cysts exposed to different concentrations of drugs *in vivo*, as determined by 8-OHdG immunofluorescence. The mice were treated with ABZ (200 mg/kg/d), AS-H (200 mg/kg/d), and AS-H+Veliparib (25 mg/kg/d). Hoechst was used for nuclear staining. Parts showing positive 8-OHdG nuclei (green). V: Veliparib.

Figure 5 shows the representative Hoechst, 8-OHdG, and 8-OHdG/Hoechst merged images of the germinal layer (GL) from cysts. Generally, no positive 8-OHdG reaction was observed in nuclei from the model group and ABZ group. The 8-OHdG positivity was observed in the GL of hydatid cysts treated with AS-H and AS-H+Veliparib, which represented the DNA damages. These results clearly suggest that oxidative damage of DNA was related to the drug intervention in *E. granulosus* hydatid cysts and PSCs.

## 4. Discussion

To our knowledge, this is the first report focusing on the effects of the combination of AS and Veliparib in cultured PSCs and the experimental Kunming mouse model. We determined the activity of PSCs after treatment using Eosin staining, which indicated that the PSCs activity decline was the most obvious in the AS-H+Veliparib group. In addition, we determined the activity of ALP in the PSCs, which indicated the activity showed significant elevation in those with severe injury. Our data showed that the activity of ALP showed an obvious increase in the AS-H+Veliparib group. On day 2, it reached the peak level, followed by a gradual decline, which may be related to the increase of PSCs death after treatment resulting in the decline in the release of enzymes.

In the *in vivo* experiments, mice were infected with CE and received AS-H and Veliparib on days 1-14, followed by AS administration via lavage after 14 days. Ultrasonography was performed to monitor the cyst volume per week during the 6 weeks. The cystic growth rate in the AS-H+Veliparib group showed a significant decline within 14 days. Compared with the AS-H group, the cyst volume in the AS-H+Veliparib group reached the lowest level at 15 days (*P* < 0.05), followed by a gradual increase of the cystic volume. Within 6 weeks after administration, the volume size in the ABZ group showed a gradual decline and reached a level of 48.57 ± 6.63% on day 25. After drug termination, the wet weight of cyst in the ABZ group and AS-H+Veliparib group showed a significant decline compared with the model group (*P* < 0.01), especially the ABZ group. No statistical differences were noticed between the ABZ group and AS-H+Veliparib group.

In the presence of oxygen, oxidation metabolism of PSCs or the immune response of the host was derived from ROS and reactive nitrogen species [31, 32]. To assess the potential effects of AS on DNA damages, comet assay based on single-cell electrophoresis was performed. Such method quantified the DNA damage as the percentage of DNA in tail. Our data showed that AS induced DNA damages in a dose-dependent manner. This implied that the ROS released by AS surpassed the natural oxygen consumption which led to oxidative stress triggering DNA damages *in vivo*. Indeed, base-excision repair was reported in the *Schistosoma mansoni* [33]. Wen et al. [18] reported a kinship between PSCs and *S. mansoni* based on the *EgRPS9* gene analysis. Therefore, the BER mechanism may exist in PSCs. On this basis, we utilized the combination of AS and Veliparib, which indicated that Veliparib could induce more severe DNA damages.

In our previous study, we firstly cloned the *RPS9* gene from the PSCs of *E. granulosus* after AS treatment [18]. It has been well acknowledged that *RPS9* gene was closely involved in the various developmental stages of the *E.g* PSCs, such as DNA repair [34]. Therefore, we tried to investigate the expression of *RPS9* gene after Veliparib treatment. Real-time PCR indicated that the expression of *RPS9* was upregulated in the presence of AS, in a dose-dependent manner. After treating with the combination of AS and Veliparib, the expression of *RPS9* gene showed downregulation compared with the AS-H group (*P* < 0.05). Western blot analysis showed that, after AS-L/H interference, the expression of *RPS9* protein was significantly higher than that of the DMSO group (*P* < 0.05). The combination of AS-H+Veliparib led to downregulation of *RPS9* compared to that of the AS-H group. In the future, further studies are required to investigate the potential mechanisms of the combination of AS and Veliparib in downregulating the expression of *RPS9* gene. According to the previous description, the activity of PARP-1 was relied on P53 as P53 could modulate the activity of RARP-1 through acetylation of SIRT1 [35]. RPS9 deficiency may activate P53, which then resulted in the apoptosis and aging process of cancer cells. Therefore, PARP-1 may be associated with the expression of *RPS9* gene. Meanwhile, the expression of *RPS9* gene was obviously upregulated in the ABZ group. To our best knowledge, ABZ could block the nutrition and glucose ingestion in the PSCs through modulating the metabolism of sulfoxide, sulfone, and 2-amine sulfone, which then resulted in glycogen depletion, together with the inhibition of the fumarate reductase. Subsequently, the generation of ATP was blocked, followed by survival and proliferation inhibition. These indicated that the pesticidal effects of ABZ may be associated with the expression of *RPS9* gene and/or protein. On this basis, more studies are required to further illustrate this.

For the limitations of this study, we cannot confirm which molecule is involved in the antiparasitic effects of AS on CE. In this study, we preliminarily investigated the molecular mechanism of AS on the CE. In the future, further studies are required to fully illustrate the exact mechanism.

In summary, we firstly reported that Veliparib could enhance the antiparasitic effects of AS on CE. Our data showed that the efficiency of AS on CE may be associated with inducing DNA damages, which then played an important role in the antihydatid cyst diseases. Our study paved the way for the research and development of drugs based on AS for treating CE.

## Data Availability

All the data were available upon appropriate request.
